# Genetic association of productive traits, carcass measurements and age at first calving in the genetic evaluation of the Nellore breed

**DOI:** 10.1007/s11250-026-05230-3

**Published:** 2026-07-23

**Authors:** F. L. Menezes, S. I. Araújo, R. B. Lobo, L. R. M. Nakabashi, F. Baldi, N. L. Pavan, G. C. I. Seidel, C. V. Araújo

**Affiliations:** 1 Núcleo de Pesquisa e Ensino em Melhoramento Animal- NUPEMA, Universidade Federal de Mato Grosso, Sinop, 78557-267 Mato Grosso Brazil; 2 Universidade Federal de Mato Grosso – UFMT, Sinop, MT Brazil; 3 ANCP - Associação Nacional de Criadores e Pesquisadores, Ribeirão Preto, São Paulo, 14020-230 Brazil

**Keywords:** Animal breeding, Beef cattle, Genetic parameters

## Abstract

The aim was to evaluate the genetic association between sexual precocity and productive performance in Nellore cattle. Records of standardized body weights at 120 (W120), 210 (W210), 365 (W365), and 450 (W450) days of age, Longissimus Muscle Area (LMA), backfat thickness (BFT), and age at first calving (AFC) were used. The mixed linear model included the fixed effects of contemporary groups (composed of herd, year, and season of birth, as well as the animal’s sex), in addition to the random effects of direct additive genetic, maternal genetic (for W120 and W210), and maternal permanent environment. The variance components were estimated by the restricted maximum likelihood method. The obtained direct heritability estimates were 0.45, 0.49, 0.42, 0.40, 0.33, 0.19, and 0.08 for W120, W210, W365, W450, LMA, BFT, and AFC, respectively. The Spearman correlations of the animals’ breeding values between the weight development estimates were of a high order, and for the traits of backfat thickness and age at first calving, it was 0.27, indicating a relationship at older ages with the onset of the acceleration of adipogenesis. The genetic gain obtained in AFC (0.48 days) with the correlated response related to LMA and BFT was − 0.05 and − 0.19 days, respectively. The genetic correlations obtained between AFC and the weight development traits were positive, however negative with the carcass-associated traits, indicating that the offspring are reproductively precocious, yet lighter and with a greater finish of subcutaneous fat.

## Introduction

Meeting the growing demand for animal protein within sustainable production systems implies the need to achieve higher productivity without expanding into new areas of exploitation. In this context, the use of selection criteria aimed at obtaining early-maturing animals becomes essential, as it reduces the production cycle and has a positive impact on production costs.

Thus, adopting selection criteria associated with weight performance that emphasize body growth can lead to higher growth rates over the total period and shorten the duration of the production cycle (Barros et al. 2018).

Coupled with the shortening of the production cycle, we improve carcass criteria by using early-maturing animals. Carcass evaluation criteria, whether obtained through a set of characteristics measured via ultrasound (May et al. 2000; Barcellos et al. 2017) or through visual scoring assessments (Lacerda et al. 2018; Freitas et al. 2020), help contribute to a better genetic expression in productive traits such as degree of muscling, fat finishing, and marbling.

Reproductive precocity in females directly impacts system profitability, as it enhances reproductive efficiency and increases the total calf output over the dam’s productive life. In genetic improvement programs, using Age at First Calving (AFC) as a selection criterion aims to improve reproductive maturity, thereby optimizing the turnover of animal categories within the production system (Ikeda et al. 2020).

Consequently, using AFC as a selection criterion leads to a reduction in rearing stages due to an increase in cows under 24 months of age that are fit for reproduction, which reduces the production cycle and intensifies the production system (Castilho et al. 2018).

Therefore, strategies that combine the simultaneous selection of productive and reproductive performance in genetic improvement programs, establishing genetic associations of sexual precocity with other (productive and carcass) selection criteria, can maximize the overall selection response (Bonamy et al., 2019).

This study aimed to estimate the genetic associations between sexual precocity traits and productive performance traits in Nellore cattle using a large and updated national database, and to evaluate the implications of these relationships for selection strategies in breeding programs.

## Materials and methods

Information was used from records of body weights standardized at 120 (W120), 210 (W210), 365 (W365), and 450 (W450) days of age, as well as records for longissimus muscle area (LMA), backfat thickness (BFT), and age at first calving (AFC) of animals participating in the Nellore Brazil Genetic Improvement Program of the National Association of Breeders and Researchers (ANCP). The data comprised animals born between 2000 and 2017.

The numerator relationship matrix included a total of 93,233 animals from 12 herds located in the states of Mato Grosso, Mato Grosso do Sul, and Goiás, which are part of the Central-West region of Brazil, one of the country’s most important beef cattle–producing regions.

Contemporary groups were formed by combining the fixed effects of herd, year and season of birth, and the animal’s sex. Two birth seasons were considered: season 1 from April to September, and season 2 from October to March. For the analyses, only information from contemporary groups with a minimum of four animals was considered, as well as sires with a minimum of four offspring.

The mixed linear model used was expressed as: **y = X*β*** **+ Z**_**a**_**+W**_**1m**_**+W**_**2p**_ **+ ε**, where **y** is a vector of observations, **X** is an incidence matrix associated with the vector **β** of solutions for fixed effects of contemporary groups and the linear and quadratic effects of the cow’s age at calving (included as a covariable, with a mean of 70.33 ± 35.91 months), **Z** is an incidence matrix associated with the vector **a** of solutions for the additive genetic random effect, **W₁** is an incidence matrix associated with the vector **m** of solutions for the maternal genetic random effect (used only for the traits W120 and W210), **W₂** is an incidence matrix associated with the vector **p** of solutions for the maternal permanent environmental random effect, and **ε** is a vector of random residuals, where, ε ~ N(0,I$\:{\sigma\:}_{e}^{2}$).

The analyses were conducted using a multitrait mixed model, in which the three traits were analyzed simultaneously to estimate the covariance structure among traits. Variance and covariance component estimates were obtained using the Restricted Maximum Likelihood (REML) method, and the Mixed Model Equations (MME) for predicting the genetic values of animals for each trait were solved using the REMLF90 program (Misztal et al. 2014).

**Table 1:** Number of records (N), mean, standard deviation, minimum and maximum values for W120, W210, W365, W450, LMA, BFT, and AFC

Traits	*N*	Mean	Standard Deviation	Minimum	Maximum
W120	59,489	136.41	16.49	100.00	180.00
W210	58,747	197.90	24.05	140.00	250.00
W365	58,658	255.51	37.57	180.00	355.00
W450	61,661	299.07	51.22	175.00	480.00
LMA	61,661	51.83	10.13	25.01	90.00
BFT	59,969	2.74	1.38	1.00	8.50
AFC	7228	35.68	7.65	20.03	54.39

Subsequently, selection efficiencies (E) were obtained as the ratios between the estimates of correlated responses (CRx/y) for gain in AFC through selection for BFT and for LMA, relative to the estimate of genetic gain (ΔGx) for AFC, expressed as: E = (RC_x/y_)/( ∆Gx), were RC_x/y_ = ix hx hy rgxy σx, in which iy = ix represents the selection intensity applied to y (with y = LMA or BFT); **hγ** is the square root of the heritability estimate of y (y = AFC); **hₓ** is the square root of the heritability estimate of x; **r_gₓγ** represents the estimate of the genetic correlation between x and y; and **σₓ** corresponds to the phenotypic standard deviation of x.

The estimate of **ΔGx** was obtained using the expression: ∆G_x_ = i_x_ σ_x_ h^2^_x_, with the same terms as described above. Using the breeding values, Pearson and Spearman correlations were obtained for all sires (1,513) with offspring in production, as well as for sires with an accuracy equal to or greater than 0.5 (141), across all traits.

Pearson and Spearman correlation coefficients were calculated among the predicted breeding values for all evaluated traits. These analyses were performed considering two groups of sires: all sires with offspring in production (*n* = 1,513) and sires with accuracy equal to or greater than 0.50 for the evaluated traits (*n* = 141). Pearson correlations were used to evaluate the linear association among predicted breeding values, whereas Spearman rank correlations were used to assess the degree of concordance in sire ranking across growth, carcass, and reproductive traits.

The Spearman correlation analysis was included as a complementary approach to the genetic correlations estimated from the multitrait mixed models. Genetic correlations describe the additive genetic association between traits at the population level, whereas Spearman correlations evaluate whether sires with favorable predicted breeding values for one trait tend to maintain favorable ranking for another trait. Therefore, Spearman correlations were interpreted only as rank-association measures among predicted breeding values, allowing the identification of possible re-ranking of selection candidates when different traits were considered simultaneously. These correlations were not interpreted as estimates of additive genetic covariance and were not used as substitutes for the genetic correlations obtained from the fitted models.

## Results

Table 1 summarizes the descriptive analyses for the traits W120, W210, W365, W450, LMA, BFT, and AFC. It was observed that for the traits related to weight performance, the magnitude of the ratio between the standard deviation and the mean body weight increased as the animals aged.

The obtained heritability estimates (Table 2) can be considered moderate to high for the weight-related growth traits, as well as for the carcass-related traits, while the estimate for AFC was of low magnitude.

**Table 2:** Heritability estimates for direct additive genetic effects (diagonal outside parentheses), maternal effects (diagonal within parentheses), and genetic correlations (above the diagonal) for P120, P210, P365, P450, LMA, BFT, and AFC

Traits	W120	W210	W365	W450	LMA	BFT		AFC		
W120	0.45(0.11)	0.91	0.86	0.83	0.32		0.01		0.31	
W210		0.49(0.06)	0.92	0.84	0.46		0.12		0.30	
W365			0.42	0.92	0.49		0.15		0.25	
W450				0.40	0.43		0.15		0.18	
LMA					0.33		0.28		-0.05	
BFT							0.19		-0.13	
AFC									0.08	

The estimated genetic gain for direct selection on AFC was 0.48 days, while the correlated responses in AFC through selection for LMA and BFT were − 0.05 days and − 0.19 days, respectively. The efficiency estimates for the correlated response of AFC expected via LMA and BFT were 0.10 and 0.40, respectively.

Genetic correlations among weight-related growth traits ranged between 0.83 and 0.92, while the correlation between carcass traits was 0.28. Positive and negative estimates were observed between AFC and weight-related performance traits, and between AFC and carcass traits, respectively (Table 2).

Pearson and Spearman correlations of predicted breeding values for all sires across the evaluated traits were estimated, followed by correlations for sires with accuracies greater than 50%, as described in Tables 3 and 4.

## Discussion

Heritability estimates for traits related to weight performance, with values of high magnitude, indicate favorable responses to the animal selection process, with the potential to promote significant genetic gains. Furthermore, the high estimates of genetic correlations, ranging from 0.83 to 0.92 among all these traits, suggest the presence of pleiotropic effects, revealing synergies in additive genetic variability for these selection criteria.

For productive traits, the heritability estimates fell within the range reported in the literature, varying from 0.25 to 0.41 (W120), 0.23 to 0.48 (W210), 0.24 to 0.29 (W365), and 0.17 to 0.52 (W450) (Ambrosini et al. 2016; Pires et al. 2016; Kamei et al. 2017; Lopes et al. 2017; Pereira et al. 2017; Souza et al. 2018; Evangelista et al. 2020; Silva Neto et al. 2020).

For pre-weaning weights, maternal heritability estimates decreased throughout this phase. This indicates reduced dependence of the offspring on the dam as this phase progresses, with the additive genetic effect becoming more significant relative to the maternal effect. Therefore, in production systems that adopt early weaning, greater attention must be paid to more intensive selection for maternal ability.

Similar estimates of maternal heritability have been reported in the literature (Ambrosini et al. 2016; Pereira et al. 2017; Silva Neto et al. 2020). However, Pires et al. (2016) reported higher heritability estimates, with magnitudes close to 0.19 ± 0.02.

For carcass-related traits, the heritability estimate for LMA (Longissimus Muscle Area) was higher (0.33), while a lower value was observed for BFT (0.19). Thus, favorable genetic gains can be expected if selection is applied to these traits. The heritability values obtained for LMA and BFT align with those reported by Buzanskas et al. (2017) and Silva Neto et al. (2020) in Nellore cattle.

The genetic correlations between AFC and the other traits, the most favorable associations were observed The heritability estimates for AFC are consistent with those observed by other authors, such as 0.09 ± 0.07 reported by Pires et al. (2016) and 0.08 by Silva Neto et al. (2020). In contrast, Buzanskas et al. (2017) found a higher estimate of 0.24.

Among all traits evaluated, AFC showed the lowest heritability estimate, indicating that most of the phenotypic variation observed for this trait was associated with environmental effects, reproductive management, data structure, and previous selection criteria applied to females, rather than being exclusively explained by additive genetic variance. This result is biologically plausible, since AFC is a female reproductive trait closely related to sexual precocity, reproductive fitness, and adaptation to the production environment. Reproductive and life-history traits, particularly those associated with fertility and early reproductive performance in females, usually show lower heritability than growth and carcass traits because they are strongly influenced by environmental conditions and have historically been exposed to natural selection (Mousseau and Roff 1987; Price and Schluter 1991). In beef cattle, these environmental effects include nutrition, age and body weight at first breeding exposure, breeding season, culling of non-productive females, and general management conditions. In Zebu cattle, this interpretation is particularly relevant. Nellore cattle originated from Bos indicus populations adapted to tropical and often restrictive environments, in which survival, rusticity, thermotolerance, parasite resistance, and reproductive ability under challenging environmental conditions are major components of biological fitness. Thus, part of the additive genetic variability related to basic reproductive adaptation may have been reduced over time by natural selection and management-driven selection. In addition, in commercial herds and breeding programs, AFC is recorded only in females that effectively calve for the first time, whereas non-exposed, culled, non-pregnant females or those that do not meet minimum developmental requirements may be excluded from the dataset. This process restricts the phenotypic variability of the trait and may contribute to reduced heritability estimates.

The estimate obtained in the present study agrees with values previously reported for Zebu populations. In Nellore cattle, Claus et al. (2017) reported heritability estimates of 0.11 for AFC and 0.13 for an alternative definition of this trait, whereas Negreiros et al. (2022) reported h² = 0.08 for AFC and highlighted that previous estimates in Nellore cattle ranged from 0.08 to 0.16. In other Zebu breeds, Freitas et al. (2022) observed low heritability for AFC in Tabapuã cattle (h² = 0.07), and Cavani et al. (2015) reported h² = 0.10 in Brahman cattle raised in Brazil. Therefore, the low heritability observed in the present study does not represent a discrepant result, but rather confirms the complex nature of this trait and its strong environmental dependence.

From an applied perspective, the low heritability of AFC indicates that the direct response to selection for reducing AFC tends to be slow when this trait is considered alone. However, this does not eliminate its importance in breeding programs, since exploitable genetic variability is still present, especially when AFC is included in selection indexes together with other reproductive and productive indicators. Considering indirect selection to reduce AFC through LMA and BFT, the estimated correlated genetic gains were − 0.05 and − 0.19 days, respectively. The relative efficiencies of the expected correlated response in AFC, based on selection applied to LMA and BFT, were 0.10 and 0.40, respectively. These results indicate that selection for carcass traits, particularly BFT, may contribute favorably, although in a limited manner, to reducing AFC. Therefore, improvement of sexual precocity in Nellore females should preferably be achieved through direct selection for reproductive traits, complemented by correlated growth and carcass criteria, and always associated with adequate nutritional and reproductive management.

Thus, the expected genetic gain from direct selection for AFC (0.48 days) indicates that this strategy is the most effective for promoting sexual precocity in females. However, carcass traits such as LMA and BFT may serve as complementary indicators in selection programs, particularly when evaluating young animals that have not yet reached sexual maturity. Although these traits can contribute to the identification of animals with a tendency for lower AFC, direct selection for AFC remains the most efficient approach to increase sexual precocity in females.

The estimated genetic correlations between weight-related growth traits and AFC indicated that selection for earlier-maturing animals would lead to lighter animals simultaneously exhibiting greater subcutaneous fat thickness.

Similar results regarding genetic correlations among reproductive, productive, and carcass traits were observed by Buzanskas et al. (2017) and Bonamy et al. (2019), who reported that sexual precocity in females would not have a negative impact on reproduction, growth, or carcass traits.

Pearson and Spearman correlations among the breeding values of all sires with offspring in production are presented in Table 3. Overall, both correlation methods showed similar patterns, indicating consistency between the linear association among breeding values and the rank-based classification of sires. High positive correlations were observed among body weight traits, especially between W120 and W210, W210 and W365, and W365 and W450, indicating that sires genetically superior for weight at one age tended to maintain favorable genetic ranking for weight at subsequent ages.

**Table 3:** Estimates of Pearson correlations (above the diagonal) among traits and Spearman correlations (below the diagonal) of breeding values for the evaluated sires for W120, W210, W365, W450, LMA, BFT, and AFC

	W120	W210	W365	W450	LMA	BFT	AFC
W120	1.00	0.91	0.86	0.82	0.22	0.13	0.10
WP210	0.90	1.00	0.93	0.84	0.42	0.18	0.07
WP365	0.83	0.92	1.00	0.93	0.45	0.22	0.04
WP450	0.80	0.81	0.91	1.00	0.33	0.29	0.04
LMA	0.21	0.40	0.43	0.31	1.00	-0.02	-0.22
BFT	0.12	0.16	0.20	0.27	-0.04	1.00	-0.23
AFC	0.13	0.09	0.07	0.07	-0.28	-0.24	1.00

The correlations between AFC and body weight traits were low, ranging from 0.04 to 0.10 for Pearson correlations and from 0.07 to 0.13 for Spearman correlations. These results indicate weak association between sire genetic merit for AFC and growth traits, suggesting limited re-ranking of sires for body weight traits when AFC is considered. In contrast, AFC showed negative correlations with carcass traits, mainly with LMA and BFT. Pearson correlations between AFC and LMA and between AFC and BFT were − 0.22 and − 0.23, respectively, while the corresponding Spearman correlations were − 0.28 and − 0.24. Considering that lower AFC is desirable, these negative correlations indicate that sires with more favorable genetic merit for sexual precocity tended to show higher breeding values for LMA and BFT.

**Table 4:** Estimates of Pearson correlations above the diagonal and Spearman correlations below the diagonal among breeding values for sires with accuracy equal to or greater than 0.50 for W120, W210, W365, W450, LMA, BFT, and AFC

	W120	W210	W365	W450	LMA	BFT	AFC
W120	1.00	0.93	0.85	0.79	0.31	-0.06	0.03
WP210	0.88	1.00	0.91	0.81	0.47	0.02	0.01
WP365	0.80	0.88	1.00	0.94	0.52	0.10	-0.06
WP450	0.76	0.77	0.94	1.00	0.46	0.18	-0.10
LMA	0.24	0.45	0.47	0.39	1.00	0.18	-0.14
BFT	-0.08	0.00	0.10	0.16	0.14	1.00	-0.30
AFC	0.02	0.00	-0.07	-0.12	-0.14	-0.27	1.00

For sires with accuracy equal to or greater than 0.50, the correlation estimates are presented in Table 4. The general pattern was similar to that observed for all sires, with strong positive correlations among body weight traits and low correlations between AFC and growth traits. This indicates that, even when only sires with greater reliability of breeding values were considered, AFC remained weakly associated with weight development. Negative correlations were also observed between AFC and carcass traits, especially BFT, with Pearson and Spearman correlations of -0.30 and − 0.27, respectively. For LMA, both Pearson and Spearman correlations with AFC were − 0.14.

These results indicate that selection for reduced AFC may be associated with a favorable, although moderate, response in carcass traits, particularly BFT. However, the low magnitude of the correlations between AFC and body weight traits suggests that selection for sexual precocity would have limited impact on the genetic ranking of sires for growth traits. Therefore, the Pearson and Spearman correlations support the interpretation that AFC can be considered in selection programs without substantially compromising growth performance, while potentially contributing to favorable changes in carcass composition.

## Conclusion

The genetic association among traits favors the selection of animals with accelerated weight gain, making them more precocious. However, selection for sexual precocity in females based on predicted breeding values of sires for Age at First Calving leads to animals with lower weight gain performance and higher subcutaneous fat content 

## Data Availability

My manuscript is associated with a database of the the Associação Nacional de Criadores e Pesquisadores (ANCP), and cannot be shared.
